# Sex Differences in Neurotoxicogenetics

**DOI:** 10.3389/fgene.2018.00196

**Published:** 2018-06-05

**Authors:** Carolina Torres-Rojas, Byron C. Jones

**Affiliations:** ^1^Department of Pharmacology, University of Tennessee Health Science Center, Memphis, TN, United States; ^2^Department of Genetics, Genomics and Informatics, University of Tennessee Health Science Center, Memphis, TN, United States

**Keywords:** neurotoxicity, sex differences, toxicogenetics, toxicants, drugs, sex susceptibility, imprinting effects

## Abstract

A major development in biomedical research is the recognition that the sex of an individual plays a key role in susceptibility, treatment, and outcomes of most diseases. In this contribution, we present evidence that sex is also important in the toxicity of many environmental toxicants and contributes to the effect of genetics. Thus, individual differences in response to toxicants includes genetic makeup, the environment and sex; in fact, sex differences may be considered a part of genetic constitution. In this review, we present evidence for sex contribution to susceptibility for a number of toxicants.

## Introduction

We are entering in the era of precision medicine, the goal of which is to make medicine more relevant to individual differences in susceptibility, treatment, and resolution of disease. Nowadays, researchers have the means to measure tens of thousands of biochemical characteristics among many animal species, including humans. These characteristics define specific phenotypes, which lead the search for underlying genes that contribute to individual differences. The contribution of sex must be considered as males and females differ in response to various pathogens.

Sex differences in neurotoxicity to several classes of chemicals, such as alcohols, heavy metals, pesticides, endocrine disruptors, and even therapeutic agents is a topic that needs to be considered to elucidate the genetic mechanisms that underlie individual differences in the effects of each toxicant. For ethical and logistical reasons research into these mechanisms is not always possible in humans and thus, we must rely on genetic reference populations of animals, most commonly, rats and mice. Common genetic animal models include knockouts-transgenics, inbred strains and recombinant inbred strains. The experimental systems genetic approach to assess host individual differences to a toxic insult involves the use of genetically defined animal models that simulate humans, this approach can help researchers to understand the role of sex and genetic differences in pathology attributable to toxicants.

When studying sex differences and genes, one might consider that sex chromosomes and related hormones are one source of differential vulnerability. Indeed, studies in experimental animals have determined that androgens can increase neurotoxicity following an insult, but the same androgens can also be neuroprotective (Legato, 2017); therefore, there is some controversy whether steroids might contribute or not to sex differences, causing vulnerability or resilience, the main point, however, consists of elucidating the mechanisms that mediate the toxic versus protective effects. For example, during brain development the hormone estradiol exerts permanent actions that can range from establishment of sex differences to pervasive trophic and neuroprotective effects.

Estradiol mediates cellular end points, including: apoptosis, synaptogenesis, dimension, and external shape of neurons and astrocytes, and physiology of the cells. All these mechanisms occurring in the brain are specific by region and depend on neuronal and glia actions and interactions (McCarthy et al., 2009).

Thus, mitochondria from the brain of female rodents have greater functional capacity, such as antioxidant and respiration functions, compared to males, (Gaignard et al., 2015). This higher specific activity of female mitochondria in brain also exists in humans (Harish et al., 2013). Differences like this from a systems genetics perspective must be correctly defined *vis-à-vis* specific neurotoxicants, as we also must be aware that many populations of neurons have distinctive biochemical phenotypes.

Here we present a short review of the literature from the past 20 years on sex susceptibility to toxicants and related biological factors that influence neurotoxicity, we resume this review in **Table 1**. Finally, according to the World Health Organization, it is necessary to mention that there is a difference between using the terms “gender” or “sex” to refer to sexual dimorphisms, the word “sex” is used to refer to biological classification, as we do in this review. Here, sex is narrowly defined as the karyotype.

**Table 1:** Sex susceptibility to toxicants and related biological factors that influence neurotoxicity.

Neurotoxicant or biological factor	Sex susceptibility	Reference	Species	Genetics or related variables	Biological characteristic
Neurochemistry	M > F	Dewing et al., 2006^1^ Beyer et al., 1991^2^	Rodent	^1^*Sry* and Tyrosine Hydroxylase. ^2^Dopamine levels	Monoaminergic system
Mitochondria	M > F	Harish et al., 2013	Human	Mitochondrial reductase, citrate synthase, and succinate dehydrogenase are higher in females	Enzymatic activity in mitochondria
Neuroimmunology and neuroepigenetics	inconclusive	Schwarz et al., 2012^1^ Nugent et al., 2015^2^	Rats	^1^Quantities of microglia are higher in males. ^2^DNA methyltransferase activity is higher in female preoptic area, 70 differentially expressed genes.	Microglia proliferation. Medial preoptic area global DNA methylation and expression.
Glutamate Glu-induced excitotoxicity	M > F	Al-Suwailem et al., 2017	Rats	Glu, GLN/GS, Glu/GABA, Glu/GLT1, GAD-67	Neurotransmitter levels. Glu induced excitotoxicity.
Medical fibrates: gemfibrozil pretreatment	M > F	Mohagheghi et al., 2013	Rats	TNF-α, NF-κB, cyclooxygenase-2, Nrf-2, superoxide dismutase, catalase, glutathione level	Antioxidant defense system and inflammatory response
Selenium	M > F	Ogawa-Wong et al., 2018	Mice	Selenoprotein P, Selenoprotein M, GSH peroxidase 1	Neurological disfunction by Selenium metabolism impairment
Nanomolar concentrations of methylmercury	M > F	Edoff et al., 2017	Primary fetal human neural progenitor cells hNPCs	*CDKL5* downregulated in male-derived cells and lower neurite outgrowth	Alteration of TUJ1-positive cells, neurite extension and cell migration, *NOTCH1* and BDNF signaling pathways
Methylmercury	M > F	Ruszkiewicz et al., 2016^1^ Woods et al., 2014^2^	^1^Mice ^2^Human	^1^TrxR, Trx and GPx. ^2^*CPOX, COMT, TDO2, GRIN2A, GRIN2B, SLC6A4, KIBRA, APOE, MT1M, MT2A, GSTT1* and *SEPP1*.	^1^Antioxidant activities. ^2^Adult cognitive performance and MeHg toxicokinetics.
Northern contaminant mixture: methylmercury, polychlorinated biphenyls, and organochlorine pesticides	inconclusive	Padhi et al., 2008	Rats	^1^*Ttr* gene upregulated in males. ^2^*Csad* and *Sparcl1* downregulated in males and *Tpi1* upregulated in females. ^3^*Pcp2* upregulated and *Tspan5* downregulated in females. ^4^*Csad* downregulated in females, *Ttr* and *CaMII* upregulated in males.	^1^Gene expression under co-exposure to toxicants and individual exposure to ^2^MeHg, ^3^polychlorinated biphenyls, ^4^organochlorine pesticides
Manganese	^∗^M > F ^∗∗^F > M	Aydemir et al., 2017^1^ Viana et al., 2014^2^ Torres-Agustín et al., 2013^3^	^1^Mice ^2,3^Human	^1^*Slc39a14*,^1∗^Manganese accumulation in the brain. ^2∗^Rey Auditory Verbal Learning *Test:* median score. ^2∗∗^Digit Span test: verbal working memory and ^3∗∗^long-term memory test scores.	^1^Transporter affecting manganese accumulation. ^2,3^Cognitive functions.
Zinc chloride and Malathion	M > F	Maris et al., 2010	*Rats*	AChE, Glutathione reductase, glutathione S-transferase, GPx and glucose-6-phosphate dehydrogenase	Cholinergic and glutathione-antioxidant systems in hippocampus and cerebral cortex
Lead	M > F	Leasure et al., 2007	Mice	Dopamine turnover in forebrain, spontaneous motor activity, amphetamine-induced motor activity and rotarod performance	Neurotoxic effects
Ethanol	F > M	Wilhelm et al., 2015)^1^ Alfonso-Loeches et al., 2013^2^ Sharrett-Field et al., 2013^3^ Butler et al., 2009^4^ Hashimoto and Wiren, 2007^5^ Devaud and Alele, 2004^6^	^1,2,5^Mice ^3,4,6^Rats	^1^*Zswim7*, *Ctgf*, *Aqp1* and *Sphk1*. TGFB1, IL-6, p38, MAPK and TP53. ^2^TLR4, iNOS and COX-2, IL-1β, TNF-α. ^3^GluN2 subunit. ^4^Pyramidal cell layer of the CA1 region of hippocampus. ^5^Cell death and DNA/RNA binding. ^6^α4 subunit content of the GABAA receptor.	^1^Genes that are targeted by glucocorticoids, immune and cell death-related responses. ^2^Levels of inflammatory mediators, apoptotic cleavage of caspase-3. ^3^Neuroadaptation of *N*-methyl-D-aspartate and GABAA receptors. ^4^Seizures in hippocampus. ^5^Ethanol-regulated genes and signaling pathways review. ^6^GABAA receptor in hippocampus and cortex.
Cocaine	^∗^F > M ^∗∗^F = M	Becker et al., 2017^1^ Pedraz et al., 2015^2^	^1^Rats ^2^Human	^1∗^Subjective effects and self-administration of the drug. ^2∗∗^Plasma cytokine concentrations. ^2∗^*N*-palmitoleoyl-ethanolamine elevated.	^1^Subjective effects. ^2^Physiological changes or immune system mobilization.
Methamphetamine	^∗^M > F ^∗∗^F > M	Cadet et al., 1997; Imam et al., 2001; Deng et al., 2002; Dluzen et al., 2003^1^ Miller and O’Callaghan, 1995; Dluzen et al., 2011^2^ Yu and Liao, 2000^3^	Mice	^1∗^Bcl-2 and PAI-1. ^2∗∗^Glial fibrillary acidic protein and insulin like growth factor 1 receptor. ^3∗^DA	^1^Factors that mediate apoptosis, thrombosis, and atherosclerosis. ^2^Protein expression. ^3^Striatal Dopamine
3,4-methylenedioxymethamphetamine, *ecstasy*	^∗^F > M ^∗∗^M > F	Pardo-Lozano et al., 2012^1^ Koenig et al., 2005^2^	^1^Human ^2^Rats	^1∗^5-HTTLPR or COMT val158met. ^1∗^Dizziness, depression/ sadness, psychotic symptoms, and sedation. ^2∗∗^Locomotor effects, hyperpyrexia, and lethality.	^1^Clinical pharmacology and behavioral effects. ^2^Physiological effects.
Organo-Phosphate pesticides: ^1^Dimethoate ^2^Mancozeb	^∗^M > F ^∗∗^F > M	Astiz et al., 2013^1^ Joode et al., 2016^2^	^1^Mice ^2^Human	^1∗^IL-6, IL-1β, TNF-α, INF-γ-inducible IP10, ERβ, steroidogenic acute regulatory protein, aromatase mRNA levels and ERα protein levels. ^2∗^Working memory. ^2∗∗^Processing speed.	^1^Expression of inflammatory molecules, production of ROS, expression of steroidogenic proteins and ER in cortical astrocyte-enriched cultures. ^2^Cognitive functions.
Organochlorine pesticides: Dieldrin	M > F	Martyniuk et al., 2013^1^ Faria et al., 2014^2^	^1^*Micropterus salmoides* ^2^Human	^1^GABAA receptor signaling in hypothalamus. ^2^Suicide rate.	^1^GABAergic and dopaminergic signaling. ^2^Behavioral effect.
MPTP	^∗^F > M ^∗∗^inconclusive	Ookubo et al., 2009^1^ Jones et al., 2013^2^ Alam et al., 2016^3^	Mice	^1∗^DA, DAT, HVA, 3,4-dihydroxyphenylacetic acid. ^2∗∗^*Mtap2*, *Lancl 1*, and *Kansl1l.*^3∗∗^DA, HVA, TH and GFAP. QTL in chr 1 at 60 Mb.	^1^Time-dependent alterations in striatum. ^2,3^Vulnerability of the nigrostriatal pathway.
Oxidative stress-induced toxicity	M > F	Giordano et al., 2013; Costa et al., 2014	Mice	*Pon2* expression is higher in females	Astrocytes and neurons sensitivity
Diesel exhaust exposure	M > F	Roqué et al., 2016^1^ Cole et al., 2016^2^	Mice	^1^Microglia mediate primary cerebellar granule neuron death *in vitro*, elevated ROS in microglia, mRNA levels of the pro-inflammatory cytokines IL-6 and IL1-β, and the release of IL-6. ^2^IL-3 and IL-6 higher PON2 mRNA in females.	^1^Microglia and cerebellar granule neurons sensitivity. ^2^Pro-inflammatory cytokines in olfactory bulb and hippocampus, oxidative stress as lipid peroxidation.
Bisphenol-A	^∗^F > M ^∗∗^M > F	Ceccarelli et al., 2007; Monje et al., 2007^1^ Kundakovic et al., 2015^2^	^1^Rodent ^2^Mice	^1∗^ERα transcription in the sexually dimorphic area of the hypothalamus. ^2∗∗^*Bdnf* at postnatal days 28 and 60, *Grin2b* at 60 days of age, amount of time spent exploring the novel environment.	^1^Effect on ER. ^2^Gene expression and behavioral effect.
Imprinting effects	inconclusive	Faisal et al., 2014	Mice	*Igf2* and *H19*	Expression levels of imprinted genes in brain


*Articles were selected for inclusion if sex differences were the topic and if significant biological processes that could be tied to genetic differences were featured as well.*

## What Are the Targets of Neurotoxicants?

### Neurochemistry and Sex Differences

Monoaminergic systems are implicated in diverse neurological pathologies and psychiatric conditions in which sex differences are seen. Serotonin, dopamine, and norepinephrine regulate several functions including respiration, neuromodulation, cognitive processes, stress responses, reproduction, and sexual behavior.

Many factors are involved in differentiation of dopaminergic neurons, including sex and genetic factors. For example, dopamine levels in diencephalic and mesencephalic cell cultures from rat embryos, even in the absence of detectable amounts of sex hormones, was shown to be higher in females compared to males (Beyer et al., 1991).

In rodents, the gene encoding the testis-determining factor, *Sry*, is present in males only, and the transcript is present in midbrain and hypothalamus of adult mouse brain (Lahr et al., 1995), Sry transcript was also observed in cortex, medial mammillary bodies of hypothalamus and substantia nigra of male rats (Dewing et al., 2006). It is also found in the midbrain of adult male mice, including the substantia nigra and hypothalamus and at all developmental stages (Mayer et al., 2000). *Sry* regulates the enzyme TH (Milsted et al., 2004; Dewing et al., 2006), the rate-limiting enzyme for catecholamine production (Molinoff and Axelrod, 1971; Nagatsu, 1995). It has been shown that the attenuation of Sry expression in rats results in detrimental motor effects and reduces the number of TH-positive neurons (Dewing et al., 2006).

The higher susceptibility of men to sporadic Parkinson’s disease (sPD), may be a consequence of greater vulnerability to toxicants that affect the expression of *Sry.* In females, estrogen is also a player as injections of estradiol benzoate in rats increase TH mRNA (Serova et al., 2004) and ovariectomy results in loss of TH-positive neurons (Leranth et al., 2000).

Estrogen regulates TH gene transcription in female and male mouse in a sex-specific manner (Thanky et al., 2002). The fact that females have smaller populations of TH neurons than males and the attenuation of Sry expression in males results in motor impairment, could be interpreted as a means by which sex exerts its influence independently of the pathway. Accordingly, this likely relates to why men have greater susceptibility to sPD than women. This could imply that this pathway is more robustly regulated in females than in males.

### Effects in Mitochondria

There are substantial sex differences in mitochondrial respiratory function in rodents and humans. Mitochondria of young females evince higher NADH-linked respiration compared to males, independently of estrus cycle. Mitochondria from the brain of female rodents have higher electron transport chain activity and ATP production and greater functional capacities (Gaignard et al., 2015). Examining the mitochondrial enzyme activities in postmortem human brains, Harish et al. (2013) observed that the enzymatic activity of mitochondrial reductase, citrate synthase, and succinate dehydrogenase were significantly higher in female compared to male human brains. In mouse brain, calcium uptake capacity was shown to be lower in mitochondria from females compared to those from males (Kim et al., 2012).

Estrogens exert protective effects on mitochondria of brain vasculature. In human brain endothelial cells, estradiol increases mitochondrial proteins and decreases ROS production. Estrogen protection against Aβ toxicity is also greater in females (Viña and Lloret, 2010), mitochondrial ROS production in neurons is higher in males (Khalifa et al., 2017). This finding may explain the higher susceptibility of males to ROS-based neurotoxicity.

### Neuroimmunology and Neuroepigenetics

Microglia are considered immunocompetent cells because of their function in immune surveillance, and their proliferation in the brain differs between females and males. In experiments with rats, after the fetal androgen surges, males, end up with significantly higher quantities of microglia in several brain regions if compared to females (Schwarz et al., 2012). Accordingly, sex differences likely involve neuroinflammatory mediators that regulate the brain development and homeostasis. This has implications for neuroimmune signaling including neural circuits of motivation, reward, and mating behavior. Additionally, microglia are an important factor for prostaglandin E_2_-mediated induction of male rat synaptic patterning in the medial preoptic area related to characteristic male copulatory behavior in adulthood. These neuroimmune sex-differences may be a cause or a factor contributing to the variations seen in neurotoxicity and drug abuse behavior because of their high influence during pathological changes in the central nervous system (McCarthy et al., 2017).

Considering neuroepigenetics, the activity of DNA methyltransferase in the preoptic area of female rats is greater compared to males, but if females are treated with estradiol, the enzymatic activity level will be reduced to that characteristic of males, but the treatment is effective only for the first few days after birth. Moreover, DNA isolated from female medial preoptic area showed greater numbers of 100% methylated CpG sites compared to males and masculinized females. Reducing DNA methylation increases the number of upregulated genes in females. Taken together with sex differences that can be reversed, this confirms that epigenetics may be one source for the sex-specific transcriptomes. Indeed, the comparison between female and male rat transcriptomes in the medial preoptic area showed only 70 differentially expressed genes from which 50% were expressed at higher levels in males whereas the other half was higher in females (Nugent et al., 2015).

### Neurotoxicity by Excitatory-Inhibitory Activity Imbalance

Glutamate (Glu) mediates excitatory synaptic transmission and this activity requires tight regulation. Glu excitotoxicity can happen via multiple mechanisms, resulting from persistent and uncontrolled neuronal activation which usually causes neuron death. For example, cell death of neurons from human embryonic stem cells has been shown under glutamate-induced excitotoxicity at the same Glu concentrations to those found in humans suffering brain injury (Gupta et al., 2013). By contrast, inhibitory neurotransmission is mediated by GABA (Marshall, 2008). The equilibrium between Glu and GABA in the brain is critically important to avoid neurological and behavioral alterations (De Witte, 2007; Gonçalves et al., 2017; Werner and Coveñas, 2017), additionally, imbalances between these neurotransmitters have been shown to be sex-specific (Higuera-Matas et al., 2012).

In a recent study, in rats, females exhibited significantly lower levels of Glu and GLN/GS, Glu/GABA and Glu/GLT1 ratios as well as lower levels of GAD-67. By comparison, the GLN/GAD-67 ratio and levels of GS were significantly higher in females than in males. Multiple regression analysis confirmed the role of the ratio GLN/GS, together with the much higher affinity of GLT1 to Glu in the clearance of Glu from the extracellular space via its uptake by various transporters helping to regulate its synaptic levels in females. Functionally, females exhibited a lower susceptibility to develop Glu-induced excitotoxicity, an etiological mechanism for multiple neurodevelopmental disorders (Al-Suwailem et al., 2017).

## Antioxidants as Anti-Inflammatory Agents: Sex Differences in Antioxidant Effects

There are some fibrates, which are fibric acid derivatives that control triglyceride levels in blood. They also have antioxidant and anti-inflammatory actions such as the peroxisome proliferator-activated receptor-alpha ligands (Fidaleo et al., 2014). A study by Mohagheghi et al. (2013) considered gemfibrozil, a fibrate that is investigated for its therapeutic actions against global cerebral Ischemia–Reperfusion injury. In this study Hippocampus tissues from male and female Wistar rats were isolated for study of inflammatory and antioxidant signaling pathways, such as nuclear factor (erythroid-derived 2)-like 2 (Nrf-2) and several endogenous antioxidant agents. When animals were exposed to the drug gemfibrozil as a pretreatment prior to the injury event, the responses were different by sex. Gemfibrozil proved to be protective for females by modulation of inflammatory factors and activation of the antioxidant defense system, including key enzymes, such as: catalase and superoxide dismutase. The drug also increased glutathione in females. In contrast, gemfibrozil pretreatment caused neurotoxic effects in males, i.e., upregulation of pro-inflammatory factors such as tumor necrosis factor-α, nuclear factor-κB, cyclooxygenase-2, decrease of Nrf-2 expression, and superoxide dismutase activity. These effects led to hippocampal neurodegeneration in males (Mohagheghi et al., 2013).

Selenium is another antioxidant (Imam et al., 1999), whose efficacy has been shown to be related to sex. Selenium is an essential trace element and has antioxidant effects that protects cells from damage and promotes male fertility. It has different functions in the brain that can vary by sex. Pitts et al. (2015) tested its action in mice castrated prepubescently in *Scly and Sepp1 homozygous knockout* mice (*Scly* is the gene encoding Selenocysteine lyase, and *Sepp1* is the gene encoding Selenoprotein P). In brain, they demonstrated that the antioxidant activity of selenium is considerably increased in castrated mice, compared to intact animals, thus providing neuroprotection. This increase reveals an existing competition between the testes and brain considering that selenium distribution is prioritized to the testes over the brain (Pitts et al., 2015).

Continuing with the sex differences related to Selenium, deletion of the Selenium transport protein (Selenoprotein P) gene in mice produces a more pronounced neurological dysfunction in males than in females. In hypothalamus, the Selenoprotein expression decreases in *Scly* homozygous knockout mice of both sexes, but males presented lower Glutathione Peroxidase 1 levels than females and the opposite was true for Selenoprotein M (Ogawa-Wong et al., 2018).

## The Toxicants

The literature for individual toxicants is summarized in **Table 1**.

### Methylmercury

Methylmercury (MeHg) is the major source of organic mercury and an environmental toxicant. Edoff et al. (2017) showed that MeHg inhibits neuronal differentiation and maturation in Human Neural Progenitor Cells, by misregulation of the single-pass transmembrane receptor (*NOTCH1*) and the BDNF signaling pathways leading to a lower number of cells containing neuron-specific class III beta-tubulin, a marker for the distribution and morphology of immature neurons (lower TUJ1-positive cells). This resulted in a noticeable reduction in cell migration and neurite extension. Sex differences showed that compared to human female fetuses, males presented the more severe alterations. Gene expression showed sex-differences in transcript abundance of *CDKL5*, by greater downregulation in male fetuses, and thus lower neurite outgrowth. This study also concluded that both gestational age of exposure to the toxicant and sex are factors influencing sensitivity.

Ruszkiewicz et al. (2016), demonstrated sexual dimorphism in mouse fetuses in response to MeHg effects on TRx, TRxR, and GPx activities. Females showed increased activity of these enzymes compared to males. TRx showed reduction in nuclear extracts from male mouse cerebellum. Gene expression analysis suggested that there is a sex-dependent post-transcriptional regulation of the antioxidant response during MeHg toxicity as evidenced by MeHg toxicity in male is characterized by the reduction of brain TRxR activity, whereas, the analysis of cytoplasmic extracts derived from female cerebrum showed a small increase in TRFXR and GX activities (Ruszkiewicz et al., 2016). These results are consistent with the general observation that females have more effective antioxidant defenses than males.

Woods et al. (2014) reported a study that included candidate genes previously related to the effects of mercury toxicity in a range that goes from neurobehavioral to mercury toxicokinetics. This range includes eight genes, *CPOX, COMT, TDO2, GRIN2A, GRIN2B, SLC6A4, KIBRA*, and *APOE* that are related to adult cognitive performance and four genes, related to mercury toxicokinetics, *MT1M, MT2A, GSTT1*, and *SEPP1*. The neurobehavioral effects were greater in boys compared to girls. When genetic variations were absent, fewer adverse sex-dependent effects were seen among children. Therefore, genetic predisposition is critical for the modulation process of the effects of mercury neurotoxicity in a sex-dependent manner in children. These genetic sex-differences that affect susceptibility may produce greater accumulation of mercury in boys than girls (Woods et al., 2014).

Padhi et al. (2008) demonstrated the effects of *in utero* and lactational exposure to the northern contaminant mixture (NCM) in rats, they designed the NCM to model the blood profile seen in Canadian arctic populations. NCM is a mixture of methylmercury, polychlorinated biphenyls, and organochlorine pesticides. They found that co-exposure to contaminants mimics the individual effects that each component alone produces, in this case, on cerebellum gene expression. As result, the *Ttr* gene was upregulated only in male rat pups. Individual exposure to MeHg caused downregulation only in males of *Csad* and *Sparcl1* genes, *Tpi1* was upregulated only in females. Individual exposure to polychlorinated biphenyls altered the expression of the genes *Pcp2* (upregulated) and *Tspan5* (downregulated) in females only. Organochlorine pesticides downregulated the expression of *Csad* in females and upregulated the expression of *Ttr* and *CaMII* genes only in males. After Propylthiouracil exposure, affected expression of *Csad*, *Sparcl1*, *Tspan5*, *Nnat*, and *Apoe* was seen only in females, while *Ttr* was upregulated only in males (Padhi et al., 2008). These results demonstrated sex differences in the cerebellum transcriptional response to individual contaminants that are masked when a co-exposure is simulated by NCM, suggesting a sex-specific risk to neurotoxicity by related environmental contaminants.

### Manganese

Manganese (Mn) is an essential micronutrient and is tightly regulated in the brain. When normal levels are exceeded, it accumulates in tissues of the central nervous system, principally in basal ganglia, therefore, is associated with symptoms similar but not identical to Parkinson’s disease, dystonia, and dopaminergic dysfunction. The Parkinsonism, dystonia and behavioral changes induced by manganese contamination has been historically reported as a consequence of higher risk exposure to the metal, namely in welders (Aschner et al., 2009).

The cell surface-localized Mn efflux transporter SLC30A10 mediates Mn efflux, reduces intracellular Mn, and protects against Mn toxicity; in general, regulates Mn homeostasis. Recently, Mukhopadhyay (2017) described intracellular functions and Mn toxicity-associated mechanism of *Slc30a10* mutants. The differences between wildtype and mutant are distinct. While in wildtype the trafficking of SLC30A10 is from endoplasmic reticulum to the cell surface, transport of Mn is from the cytosol to the cell exterior, protecting against manganese toxicity. In the mutants, SLC30A10 remains trapped in the endoplasmic reticulum and thus cannot effect Mn efflux, increasing the levels of intracellular Mn and therefore toxicity.

Another manganese transport defect occurs when there are mutations in the zinc transporter gene *SLC39A14*. These mutations produce an excessive accumulation of manganese producing Parkinsonism and dystonia effects in onsets during childhood, even causing neurodegeneration as corroborated in post-mortem examinations (Tuschl et al., 2016).

Aydemir et al. (2017), showed that zinc transporter ZIP14 (encoded by *SLC39A14* gene) is essential for Mn elimination via the gastrointestinal tract. In animal models, deletion of *Slc39a14* results in Mn accumulation in the brain. Mn accumulation in brain also increases with age, which is consistent with the progression of signs of Parkinson’s disease with age. These researchers also showed manganese accumulation to be greater in male mice compared to females.

Results from studies examining exposure to manganese could be translated into behavioral effects as shown in a study by Torres-Agustín et al. (2013), where they found a negative correlation between manganese in hair and long-term memory test scores, resulting the girls showing the greater Mn-related effect. Another study by Carvalho et al. (2014), found that verbal immediate and working memory scores were negatively associated with *Mn in hair*, indicating prefrontal cortex and fronto-striatal circuit impairment, however, they also reported that “age and mother’s formal education level were confounding variables” (Carvalho et al., 2014).

Another behavioral report is the result from the Rey Auditory Verbal Learning Test, in which the median score was significantly higher for women than for men, but in the verbal working memory test, men had a better median score on the Digit Span test than women (Viana et al., 2014). Children that have high *Mn in hair* present cognitive impairment, mostly in the “verbal domain.” The education level of children representatives or caregivers is inversely correlated with Mn exposure, suggesting that, children’s cognition levels may be susceptible to manganese exposure either by direct or indirect mechanisms, however, there were no significant differences by sex (Menezes-Filho et al., 2011).

### Zinc Chloride and Malathion

The measurement of biochemical parameters related to cholinergic and glutathione-antioxidant systems in brain regions are important to elucidate the critical enzymes that function in a sex-specific manner under Malathion neurotoxicity.

Investigators treating rats with Malathion, reported pronounced reduction of acetylcholinesterase activity, glutathione S-transferase and glutathione reductase in the hippocampus and cerebral cortex in rats with males being more susceptible than females (Maris et al., 2010).

Similarly, zinc chloride treatment produces a decrease in acetylcholinesterase activity in both male and female cerebral cortex. Enzyme activity specific to male antioxidant defenses was attenuated, more specifically, activity for cortical glutathione reductase, glutathione S-transferase, GPx and glucose-6-phosphate dehydrogenase; and hippocampal glutathione reductase (Maris et al., 2010). This effect may be reversed by zinc treatment as seen in a previous study in adult male Wistar rats that applied oral zinc as pretreatment and achieved a completely reversible effect of malathion-induced impairment on rat hippocampus antioxidant defenses (Franco et al., 2009). Further genetic studies may be done to elucidate how these sex variants are related to differences in gene expression.

### Lead

Lead concentrations in blood tend to be higher in men than in women (Kim et al., 2006; Choi et al., 2017). Periods of high bone turnover increase absorption of endogenous lead, particularly in women during pregnancy and lactation (Manton et al., 2003). Heavy metals such as mercury and lead, are highly transferable from mother to fetus when they are in the forms of mercury vapor and methylmercury. This early exposure produces toxic effects to the fetus and studies show that boys are more susceptible to neurotoxic effects, while females are more susceptible to immunotoxicity of lead (Vahter et al., 2007). The difference in immunotoxicity may be related to the sex-differences in the quantities of microglia, which is related to immune surveillance, as we mentioned in the section “Neuroimmunology and Neuroepigenetics” these quantities are higher in males.

Lead exposure during gestation in C57BL/6 mice, produced persistent increased dopamine turnover in forebrain, decreased spontaneous motor activity, increased amphetamine-induced motor activity, and decreased rotarod performance in year-old male mice if compared to females (Leasure et al., 2007).

### Ethanol

Wilhelm et al. (2014) using alcohol withdrawal seizure-resistant mice and withdrawal-seizure-prone mice demonstrated strain-specific pathways of inflammatory signaling following at peak ethanol withdrawal, with a pro-inflammatory action in females but a marked suppression of immune signaling in males. In 2015, the same authors used withdrawal seizure-resistant mice only and collected tissue for gene expression from the medial prefrontal cortex, frontal association cortex, prelimbic cortex, infralimbic cortex, rostral portions of secondary motor cortex, anterior cingulate cortex, primary motor cortex, medial, ventral, and lateral orbital cortex, agranular insular cortex, and dorsolateral orbital cortex. The results showed a number of astrocytic genes that were regulated in a sex-specific fashion. These included *Ctgf*, *Sphk1*, and *Aqp1*. Also, *Zswim7*, that controls the homologous recombination repair pathway in early stages when double-stranded DNA breaks occur during DNA-replication or DNA-repair after insult by damaging agents, was highly up-regulated by ethanol in males compared to females, during the peak of ethanol withdrawal (8 h after chronic intoxication) and during abstinence (21 days after withdrawal) (Wilhelm et al., 2015).

In general, in females ethanol exposure causes greater up-regulation of several genes that are targeted by glucocorticoids, while males displayed a more balanced pattern of up and down regulation. Females presented activation of corticosterone response, transforming growth factor β1, IL-6, p38 mitogen-activated protein kinase, and TP53, which are regulators related to immune and cell death systems, consistent with the observed cell death at peak withdrawal in the medial prefrontal cortex (Wilhelm et al., 2015).

Another study with C57BL/6 wildtype and *Tlr4* knockout mice has shown that ethanol activates the innate immune system by acting as an agonist of TLR4 in glial cells, activating *Tlr4* signaling and producing inflammatory mediators, such as iNOS and COX-2 along with cytokines interferon 1beta, TNF-α and IL-6. Ultimately, this cascade is responsible for neuroinflammation, brain injury, white matter impairment, and finally, neurodegeneration (Alfonso-Loeches et al., 2013).

The role of TLR4 has been further demonstrated by showing that chronic ethanol intake does not cause neuroinflammation, demyelination, and brain damage in TLR4-deficient mice. Chronic ethanol consumption up-regulates inflammatory mediators (iNOS and COX-2) and cytokines (IL-1β and TNF-α), increases caspase-3 cleavage in the cerebral cortex of both female and male mice, and that the levels of inflammatory mediators, and even the apoptotic cleavage of caspase-3, tend to be higher in female than in male mice. The levels of caspase-3 were significant higher in females than in males. (Alfonso-Loeches et al., 2013).

Devaud and Alele (2004), demonstrated that male rats show greater levels of α4 subunit content of the GABAA receptor in both hippocampus and cortex following prolonged alcohol exposure, as compared to females.

There are sex differences in the neuroadaptation of *N*-methyl-D-aspartate and GABAA receptors to alcohol and sex differences in ethanol seizure susceptibility. Female rats were more susceptible than male rats and the mechanism was reported to be tied to sex differences in the GluN2 subunit that modulates polyamine spermidine in the hippocampus (Sharrett-Field et al., 2013). Also, compared to males the pyramidal cell layer of the CA1 region of hippocampus in female rats exhibits greater seizure activity during alcohol withdrawal and may be modulated by the adenosine A1 receptor (Butler et al., 2009).

In female mice alcohol effects are more pronounced than in males via dysregulation of genes related to cell death and DNA/RNA binding mechanisms, while other variables are impaired in males such as protein degradation, and calcium ion binding pathways. Overall, the research in genetics to elucidate ethanol-regulated genes and signaling pathways shows higher neurotoxicity in females, compared to males (Hashimoto and Wiren, 2007). These investigations support the view that women are more susceptible to the medical outcomes of alcohol abuse, suggesting that females are more vulnerable than males to the neurotoxic and neuroinflammatory effects of ethanol.

### Drugs of Abuse

There are subjective effects in women caused by drugs of abuse consumption that are more intense during the follicular phase of the menstrual cycle and this may be related to elevations in circulating estradiol (Becker et al., 2017).

Cocaine, amphetamine, and methamphetamine are readily self-administered intravenously by rats. Compared to intact females, ovariectomized rats evince decreased cocaine self-administration, an effect that is reversed by estradiol treatment compared with males (Becker et al., 2017). These results evidence the influence of estradiol stablishing sex differences in the consumption of drugs of abuse.

In long-time cocaine users of both sexes, Pedraz et al. (2015), evaluated plasma cytokine concentrations, including pro-inflammatory and anti-inflammatory mediators, and found that IL-1β, IL-6, IL-10, and TNF-α were higher in healthy women compared to men, but these differences were not present in long-term cocaine users of both sexes. The inference is that cocaine abuse may attenuate sex-differences in the immune system. Additionally, the investigators reported an increase, only in cocaine-addicted women, of the naturally occurring lipid *N*-palmitoyl-ethanolamine, an anti-inflammatory fatty acid.

Methamphetamine (MA) produces significant alterations in factors that mediate apoptosis, thrombosis, and atherosclerosis, such as B-cell lymphoma 2 (Bcl-2) (Cadet et al., 1997; Imam et al., 2001; Deng et al., 2002; Dluzen et al., 2011) and plasminogen activator inhibitor-1 in male mice (Dluzen et al., 2003), in the case of Bcl-2, downregulation and upregulation of expression after methamphetamine doses is dependent of the timing when measurement is done. Different proteins were altered only in females, i.e., an increase of GFAP (Miller and O’Callaghan, 1995; Dluzen et al., 2003) and a decrease in the insulin like growth factor 1 receptor (Dluzen et al., 2003, 2011). Experiment with mice carrying a heterozygous mutation in the dopamine transporter, shows that only female mice presented significant alterations in striatal dopamine transporter binding and mRNA (Ji et al., 2009) leading to lower concentrations in striatal dopamine along with higher output of striatal dopamine induced by methamphetamine (Ji and Dluzen, 2008).

In BALB/c and C57BL/6J mice of both sexes, female mice of both strains displayed the lowest depletion of striatal dopamine with MA treatment at proestrus (estrogen levels increase), whereas the greatest depletion of striatal dopamine was observed with MA treatment during diestrus (estrogen levels are variably low and progesterone levels increase). In the C57BL/6J strain, males exhibited greater striatal dopamine depletions compared to females. But, male and female BALB/c mice demonstrated a similar dopamine depletion in striatum following MA treatment. When comparing between strains, regardless of sex, C57BL/6J mice demonstrated around 1.4 to 2.2 times greater striatal dopamine depletion compared to BALB/c mice (Yu and Liao, 2000). Taken together, these findings of sex-differences between mice strains suggest the relevance of the modulating factors related to drug metabolism, besides of sexual hormones, playing a role in striatal dopamine regulation under MA insult.

3,4-Methylenedioxymethamphetamine or MDMA is a synthetic psychostimulant that has effects on neurotransmission, for example producing enhanced striatal serotonin and dopamine outflow (Rizzo et al., 2018). It also inhibits cytochrome P-450 2D6 (Yubero-Lahoz et al., 2011). Pardo-Lozano et al. (2012) studied the subjective effects of MDMA in users. The negative effects observed were dizziness, depression, sadness, and sedation. Moreover, women experienced more adverse cardiovascular and subjective effects than men. The important finding was that some of the negative effects were modulated by genotypes related to females and polymorphisms in the serotonin-transporter or polymorphism of the gene encoding catechol-*O*-methyltransferase (Val^158^Met). This agent may also produce greater depletion of serotonin in women compared to men (Pardo-Lozano et al., 2012). Previously, Koenig et al. (2005), reported results of two experiments in pubescent Long-Evans rats. In experiment 1 all males died after the third injection of MDMA and surviving females showed serotonin depletion in hippocampus and cortex. In experiment 2, males demonstrated higher sensitivity to MDMA, showing greater locomotor effects, hyperpyrexia, and lethality than females.

### Pesticides: Organophosphate Compounds, Fungicides, Insecticides, Herbicides

Presently there is growing evidence that males are more susceptible than females to the adverse effects of pesticides, except for some effects on motor function preferentially affecting females.

Experimentation *in vitro* with astrocytes from postnatal day 1 mouse pups and exposing these cultures to the organophosphate insecticide dimethoate, showed sex differences in levels of ROS, IL-6, interleukin 1 beta, TNF-α, IP10, estrogen receptor beta, steroidogenic acute regulatory protein, aromatase mRNA, and ERα protein where males exhibited higher levels than females (Astiz et al., 2013). These investigators then cultured neurons from males in the presence of estradiol and showed deceases in transcripts of the genes encoding IL-6, IP10, TNF-α, and IL-1β. Moreover, ROS production in astrocytes of males was also reduced to female levels (Astiz et al., 2013). This is a direct evidence of estrogen protection against some neurotoxicants.

The factors that determine the effects and characteristics of exposure make the outcomes highly variable even for the same organophosphate compound. These factors were defined by Comfort and Re (2017) as the type of species for the sample, age of individuals, neurological characteristics that will be assessed, areas of the brain, cell types, type of behavioral assay employed, the dose, route of administration, type of chemical compound, among others.

Certain pesticides may affect children’s neurodevelopment. Joode et al., 2016 found that in children (6- to 9-years-old) living near banana plantations and plantain farms in the Talamanca County, Costa Rica and exposed to Mancozeb, a Mn-based fungicide showed impaired neurobehavioral traits. Levels of exposure were determined by analyzing urinary metabolites of chlorpyrifos (TCPy), mancozeb (ethylenethiourea), and pyrethroids (3-phenoxybenzoic acid, 3-PBA) (Joode et al., 2016). These investigators reported that compared to girls, boys showed greater urinary TCPy concentrations and poorer working memory in boys. Higher levels of 3-PBA and poorer processing speed scores were observed in girls.

Dieldrin is an organochlorine pesticide found in aquatic areas as a pollutant. Vertebrate animals exposed to this toxicant generally develop neurological and reproductive problems.

Martyniuk et al. (2013) showed neurotoxic effects of dieldrin in female and male largemouth bass (LMB), an effect attenuated by E2 in males. Mechanistically, dieldrin’s negative effects were observed in GABAergic and dopaminergic signaling; GABAA receptor signaling in hypothalamus was impaired in males. These results show one more time the protective potential of E2 and the regulation of steroidogenic-factor-1 expression networks as sex-dependent mechanisms that are meaningful translated to sex differences in onset of neurodegenerative diseases in humans.

Also, females had a significantly higher gonadosomatic index compared to males, perhaps explaining why males treated with dieldrin and E2 had more similar gene expression compared to males treated with dieldrin only than with the female group. Female LMB also had significantly more gene transcripts affected by dieldrin than males (Martyniuk et al., 2013).

Further investigation of transcript changes revealed that 561 hypothalamic genes were highly correlated and common for both females and males exposed to dieldrin, suggesting a sex independent pathway for this subset of genes. Exposure to dieldrin increased expression levels in female hypothalamus of genes related to the signaling pathways of vasopressin receptor 1, dopamine receptor 2, tumor necrosis factor receptor, nerve growth factor receptor, and alteration of gene expression that targets the signaling of GnRH, retinoic acid receptor, thyrotropin-releasing hormone, and dopamine receptor D1 (Martyniuk et al., 2013). In males, dieldrin decreased activity in apoptosis regulation and in several signaling pathways that are receptor-mediated for follicle stimulating hormone, B-cells, interleukin-7, angiopoietin, growth hormone, as well as, were affected signaling of GnRH, androgen receptor, nuclear receptor coactivator 2, follistatin, GABAA receptor, and dopamine receptor D2 (Martyniuk et al., 2013). In males exposed to dieldrin and E2, growth hormone signaling, protein tyrosine phosphatase, receptor type, C signaling cascade, and the serotonin receptor signaling were all increased (Martyniuk et al., 2013).

Behavioral consequences of pesticide exposure show sex differences as well as demonstrated by Faria et al. (2014), who reported dieldrin exposure versus suicide rate in an area of Brazil, in the period between 2006 and 2010. The incidence rate of suicide was 6.4 events per 100,000 person-years, in where men presented the higher prevalence over women (male/female ratio of 4.2) (Faria et al., 2014).

### MPTP: Parkinson’s Disease Model

The proneurotoxicant, MPTP is metabolized to MPP+ that targets the nigrostriatal pathway by destroying TH-containing neurons in the substantia nigra, pars compacta. MPTP is used in animals to model the course of Parkinson’s disease. Time-dependent alterations in striatum and sex differences were observed in this PD model, levels of dopamine, dopamine transporter, homovanillic acid, 3,4-dihydroxyphenylacetic acid were lower in female mice compared to males (Ookubo et al., 2009).

An initial study using ten different strains of male BXD mice found large strain-related differences in MPTP-related damage to striatal dopamine neurons (Jones et al., 2013). Additionally, these investigators showed that transcripts representing the genes *Mtap2*, *Lancl 1*, and *Kansl1l* were strongly correlated with loss or damage of striatal dopamine neurons.

A follow-up study by these investigators in females from the same strains reported significant agreement between both sexes for the effect of MPTP on Dopamine, Homovanillic acid, and TH. Although, both sexes presented highly similar responses to MPTP insult, they found some differences, notably in the production of GFAP, a marker of astrocyte activation. Mapping of the index for dopamine damage in the striatum yielded the same QTL on chromosome 1 at 60 Mb (Alam et al., 2016).

### Air Pollutants and Diesel Exhaust Exposure

Diameter is a characteristic of airborne particulate matter related to its toxicokinetics. Of great concern is ultrafine particulate matter, as these particles can enter the circulation system very easily and distribute systemically, even to the brain. Air pollution is believed to exert its peripheral toxicity by oxidative stress and inflammation.

The air pollutant related to vehicle traffic contains diesel exhaust (DE) (Ghio et al., 2012), which is composed of ultrafine particulate matter. Paraoxonase-2 may be protective under exposure to DE in a sex-dependent fashion. Greater *Pon2* expression has been seen in females compared to males in mice, possibly because this enzyme is modulated by estrogens (Giordano et al., 2013; Costa et al., 2014).

The neuroinflammation hypothesis of air pollution is that the particles elevate proinflammatory cytokines and ROS in the brain, thereby mediating the neurological damage produced by air pollution. Roqué et al. (2016) showed that microglia and cerebellar granule neurons in male mice have higher sensitivity to neurotoxicity by exposure to DE particulate matter if compared to females, and they suggested that this difference may be caused by the lower paraoxonase-2 expression in males. Moreover, the MAC1-NOX2 pathway in microglia appears to be a major player (Jayaraj et al., 2017).

Giordano et al. (2013) used C57BL/6J *Pon2* knockout mice (vs. wildtypes) of both sexes, for quantification of cell viability. *Pon2* encodes a protein that is widely expressed and acts as a cellular antioxidant. The experiments *in vitro* and *in vivo* demonstrated that estrogens are significant modulators of PON2. The PON2 mRNA and protein levels, as well as, lactonase activity were higher in female mice brain and cells. Astrocytes and neurons from males showed greater sensitivity to oxidative stress than female cells. *Pon2* knockout mice did not show sex-differences in susceptibility after exposure to the same oxidants. Estradiol treatment showed greater increase of PON2 mRNA and protein levels in male astrocytes, and such increase was dependent on the activation of estrogen receptor alpha. Estradiol protective effects were seen in astrocytes from wildtype mice but not from *Pon2* knockout mice (Giordano et al., 2013). These results suggest that PON2 is a major intracellular factor that interacts with estradiol to exert protection to central nervous system cells against oxidative stress and such protection is lower in males due to the lower levels of PON2 expression in brain regions, which may lead to higher susceptibility to neurotoxicity and, therefore, to neurodegenerative diseases.

One would expect, therefore, that females would show greater resistance to the neurotoxic effects of DE than males. In fact, increases in pro-inflammatory cytokines in olfactory bulb and hippocampus after DE exposure were observed to be higher in males compared to females (Cole et al., 2016). Sex difference in *PON2* expression across species are also observed, with female mice, rats, humans, non-human primates displaying higher *PON2* mRNA, protein, and activity levels (Costa et al., 2013; Giordano et al., 2013), all attributable to the influence of estrogens on PON2 gene expression (Giordano et al., 2013). Thus, *in vitro* studies on different types of brain cells, i.e., neurons, astrocytes, and microglia from male mice demonstrated higher susceptibility to toxicity compared to cells from female mice that were exposed to DE (Giordano et al., 2013).

Glutamine-cysteine ligase is the rate-limiting step in glutathione production, a major antioxidant. Age, appeared to modulate air pollution neurotoxicity. The experiment, contrasted the effects of acute DE exposure on cytokine levels in hippocampus and olfactory bulb of homozygous *Gclm*, heterozygous *Gclm* and knockout *Gclm* mice, where IL-3 and IL-6 were increased upon DE exposition, these interleukins appeared to be more affected in males than in females (Cole et al., 2016). These results highlight the relevance of considering the analysis by sex and age, as well as, the influence of genetic polymorphisms that may increase susceptibility to traffic-air pollution, when investigating in the neurotoxicogenomics field.

### Hormone Disruptors

Endocrine disruptors are found in many household and industrial products. These agents affect the natural processes related to cell metabolism where hormones are involved, such as: homeostasis, development, and fertility. In their review, Palanza et al. (2016) mention that cerebral cortex, hypothalamus, and hippocampus of rodent brain are affected by exposure to endocrine disruptors during prenatal and perinatal stages producing effects that differ by sex.

Bisphenol-A (BPA) is an estrogenic endocrine disruptor that has negative effects on luteinizing hormone and testosterone that may cause behavioral problems similar to those seen in schizophrenia, particularly, in males who attempt suicide, luteinizing hormone was marginally elevated, and testosterone decreased (Tripodianakis et al., 2006). Unfortunately, humans are frequently exposed to BPA, because it is used in plastic, resins, and other products, and virtually everyone has detectable BPA in blood (Vandenberg et al., 2007).

In rodents, the effect of BPA on ER is dependent on dose, region, and sex. Exposure to BPA neonatally and pre-puberty disrupts ER-alpha transcription in the sexually dimorphic area of the hypothalamus in females but not in males (Ceccarelli et al., 2007; Monje et al., 2007), this deregulation may also affect the anteroventral periventricular nucleus in preoptic area of hypothalamus (Monje et al., 2007).

In neonatal female rats, exposure to BPA increases ER-alpha but not of ER-beta in medial basal hypothalamus but not in the anterior pituitary. In contrast, in males, BPA increases ER-alpha and -beta in the pituitary but not hypothalamus (Khurana et al., 2000). According to these authors, these effects in ERs may be explained partially by delayed but sustained hyperprolactinemia preweaning in both sexes (Khurana et al., 2000).

Bisphenol-A has also been shown to impair oxytocin-dependent maternal behaviors, such as arched back posture and licking-grooming (Della et al., 2005).

In mice exposed to BPA *in utero* persistent DNA methylation in the gene that encodes for BDNF occurs in the hippocampus and blood. Investigators examined the *Bdfn* and NMDA receptor 2b subunit (*Grin2b*) genes associated with neuronal plasticity. These genes undergo epigenetic dysregulation in response to exposure to BPA. Expression of BDNF mRNA was significantly decreased in males and was significantly increased in females, and *Gadd45*b expression was decreased in males. These results suggested that DNA hypermethylation may be driving the BDNF down-regulation in males because the role of *Gadd45b* in the DNA demethylation of the BDNF gene. Prenatal BPA exposure, produced a decrease in the time spent exploring a novel environment in males compared to females (Kundakovic et al., 2015). The authors attributed these differences to changes observed in BDNF gene expression at postnatal days 28 and 60, as well as to down-regulation of *Grin2b* in males at 60 days of age.

Bisphenol-A was shown to enlarge the locus coeruleus in males to sizes comparable to those in females, which have normally larger loci. This demonstrates a sex difference attenuated by a toxicant (Masuo and Ishido, 2011).

## Imprinting Effects on Neurotoxicogenomics

Patten and Haig (2008) described the modeling of two different patterns of imprinting. Imprinting occurs when gene expression is dependent on the sex of the donor parent. For example, if the gene from the male parent has been silenced by methylation, then the maternal copy of the gene will be expressed. This is an example of epigenetics. This means that offspring carrying identical genotypes at the imprinted locus may show very different phenotypes.

Faisal et al. (2014) studied C57BL/6J/129-mixed background mice. The results showed sex-differences in the expression of the following genes: *Peg3, Zim1, Igf2, H19*, and *Zac1. Peg3* is the paternally expressed gene 3, *Zim1* is zinc finger imprinted 1, *Igf2* is insulin-like growth factor 2, *H19* is H19 mRNA, *Zac1* is zinc finger protein 1 regulating apoptosis pathway and cell cycle arrest. The expression levels of this set of imprinted genes in brain are usually higher in males compared to females. However, only the genes *Igf2* and *H19*, showed statistically significant differences. The precise mechanisms that model these sex differences in gene expression are still unknown (Faisal et al., 2014).

## Conclusion

Considering that individual differences affect the extent of susceptibility to the effects of toxicants, one important aspect is contribution of sex. We have summarized (**Table 1**) important studies about the matter, even when there is not much information with the combination sex and gene-dependent neurotoxicity, we know for sure that every toxicant exerts an action that depends on the configuration of its biological mechanism., Yet, at the same time, such mechanisms are determined by a *key matrix* which is the genetic constitution. In this way, because the gene SRY is not present in females and has been demonstrated that its down-regulation results in detrimental motor effects in males, it has been shown that in males SRY regulates the transcription of the enzyme TH, while in females estrogens regulate it. Moreover, it is also different the mitochondrial respiratory function, in which female function is more efficient, in steroid mediated transcription and levels of glutamate, males are more susceptible than females. Medical drugs like the fibrate Gemfibrozil could be therapeutic in females but toxic to males. It has been shown that males are more susceptible to different toxicants like methylmercury, manganese, lead, MTT, DE, and imprinting effects as well, in contrast, females are more susceptible to ethanol and some drugs of abuse like cocaine. However, there are still undetermined effects for some toxicants like methamphetamine, pesticides, and hormonal disruptors. Thus, females may be the most negatively affected or not. These outcomes depend on the design of the experiment, what function is being evaluated, brain area, hormones, enzymes, pathways, metabolites, neurotransmitters, or behavioral parameters.

## Author Contributions

All authors listed have made a substantial, direct and intellectual contribution to the work, and approved it for publication.

## Conflict of Interest Statement

The authors declare that the research was conducted in the absence of any commercial or financial relationships that could be construed as a potential conflict of interest.

## Abbreviations

ATP: adenosine triphosphate
*Apoe*: apolipoprotein E
*Aqp1*: aquaporin-1
BDNF: brain-derived neurotrophic factor
BPA: Bisphenol-A
CA1: cornu ammonis area 1 of hippocampus
*CaMII*: calmodulin II
*CDKL5*: cyclin-dependent kinase-like 5
*COMT*: catechol-O-methyltransferase
*CPOX*: coproporphyrinogen oxidase
*Csad*: cysteine sulfinic acid decarboxylase
*Ctgf*: connective tissue growth factor
E2: Estradiol
ER: estrogen receptors
GABA: gamma aminobutyric acid
GABAA: gamma aminobutyric acid receptor
GAD-67: glutamate decarboxylase-67
*Gadd45b*: DNA damage-inducible beta gene
*Gclm*: glutamate-cysteine ligase modifier subunit
GFAP: glial fibrillary acidic protein
Gln: glutamine
GLN: glutaminase
GLT1: glutamate transporter 1
Glu: glutamate
GluN2: glutamate receptor GluN2 subunit
GnRH: gonadotropin-releasing hormone
GPx: glutathione peroxidase
*GRIN2A*: glutamate ionotropic receptor NMDA type subunit 2A
*Grin2b*: NMDA receptor 2b subunit gene
GS: glutamine synthetase
*GSTT1*: glutathione S-transferase theta 1
*H19*: gene for a long non-coding RNA
*Igf2*: insulin-like growth factor 2 gene
IL-1β: interleukin 1 beta
IL-3: interleukin-3
IL-6: interleukin-6
IP10: interferon-γ-inducible protein 10
*Kansl1l*: KAT8 regulatory NSL complex subunit 1 like gene
*KIBRA*: protein phosphatase 1, regulatory subunit 168
*Lancl 1*: (Bacterial lantibiotic synthetase component C)-like 1 gene
MAC1: microglial integrin receptor
MDMA: 3,4-methylenedioxymethamphetamine, ecstasy
MeHg: methylmercury
MPTP: 1-methyl-4-phenyl-1,2,3,6-tetrahydropyridine
*MT1M*: metallothionein 1M
*MT2A*: metallothionein 2A
*Mtap2*: microtubule associated protein 2 gene
NADH: nicotinamide adenine dinucleotide reduced
NADPH: nicotinamide adenine dinucleotide phosphate
NMDA: *N*-methyl D-aspartate
NOX2: NADPH oxidase
*Pcp2*: purkinje cell protein 2
*Peg3*: paternally expressed gene 3 protein
PON2: paraoxonase/arylesterase 2
QTL: quantitative trait loci
ROS: reactive oxygen species
*Scly*: Selenocysteine lyase
*Sepp1*: Selenoprotein P
*SLC6A4*: Solute carrier family 6 member 4
*Sparcl1*: SPARC-like 1
*Sphk1*: sphingosine kinase 1
TCPy: 3,5,6-trichloro-2-pyridinol
*TDO2*: tryptophan 2,3-dioxygenase
TH: tyrosine hydroxylase
TLR4: toll-like receptor 4
TNF-α: tumor necrosis factor alpha
TP53: tumor protein P53
*Tpi1*: triosephosphate isomerase 1
Trx: thioredoxin
TrxR: thioredoxin reductase
*Tspan5*: tetraspanin 5
*Ttr*: transthyretin
*Zac1*: potential tumor suppressor gene
*Zim1*: imprinted zinc-finger gene 1
Zswim7: zinc finger SWIM-type containing 7
